# Transfer of the Active Ingredients of Some Plant Protection Products from Raspberry Plants to Beehives

**DOI:** 10.1007/s00244-017-0488-4

**Published:** 2017-12-15

**Authors:** Bartosz Piechowicz, Ewa Szpyrka, Lech Zaręba, Magdalena Podbielska, Przemysław Grodzicki

**Affiliations:** 1 Department of Analytical Chemistry, Institute of Biotechnology, University of Rzeszów, Werynia, Poland; 2 Laboratory of Pesticide Residues, Institute of Plant Protection, National Research Institute, Rzeszów, Poland; 3 Faculty of Mathematics and Natural Sciences, University of Rzeszów, Rzeszów, Poland; 4 Department of Animal Physiology, Faculty of Biology and Environmental Protection, Nicolaus Copernicus University, Toruń, Poland

## Abstract

Plant protection products (PPPs) have been found increasingly in the environment. They pose a huge threat to bees, contributing to honeybee colony losses and consequently to enormous economic losses. Therefore, this field investigation was designed to determine whether their active ingredients (AIs) were transferred from raspberry plants to beehives located in the immediate neighbourhood of the crop and to what extent they were transferred. Every week for 2 months, samples of soil, raspberry leaves, flowers and fruits, worker bees, honeybee brood, and honey were collected and analysed for the presence of propyzamide, chlorpyrifos, iprodione, pyraclostrobin, boscalid, cypermethrin, difenoconazole, azoxystrobin, and pyrimethanil residues. Five of these substances were found in the worker bee bodies. Chlorpyrifos, applied to only the soil through the irrigation system, also was detected in the brood. A small amount of boscalid was noted in the honey, but its residues did not exceed the maximum residue level. For chlorpyrifos, boscalid, and pyrimethanil, a positive correlation between the occurrence of PPPs in the crops and the beehives was found. Statistical methods confirmed that the application of PPPs on a raspberry plantation, as an example of nectar-secreting plants, was linked to the transfer of their AIs to beehives.

The honeybee (*Apis mellifera* F.) is an insect species of significant importance to the biosphere and the economy (Free 1993; Delaplane and Mayer 2000). This pollinator influences the yields of approximately 70% of cultivated plants, which represents approximately 35% of the total global food production (Klein et al. 2007), which, in turn, yields $150 billion per year. In Brazil, the value of the work performed by all pollinators is estimated at nearly $ 12 billion (Giannini et al. 2015). In Great Britain, for Gala apples, the value of bees as pollinators is estimated at £5.7 million a year (Garratt et al. 2014). Majewski (2014) showed that a decrease in the number of the honeybee colonies in Poland caused a decline in total crops valued at approximately €728.5 million. In general, the profit earned through pollination by bees is approximately 25–30% of the total yield of the crop (Sanjerehei 2014; Giannini et al. 2015). According to Sanjerehei (2014), this value is 54 times higher than the value of honey produced by bees. *A. mellifera* is the main pollinator that generates 86.8% of the gains generated by all the pollinators. The use of plant protection products (PPPs) on nectar-secreting plants goes hand in hand with the problem of exposing pollinators to such substances (Piechowicz et al. 2018a, b).

PPPs may enter hives due to foraging by worker bees (Balayiannis and Balayiannis 2008; Mao et al. 2013; McMenamin and Genersch 2015), because their active ingredients (AIs), especially those that have a contact activity, are present in crops, and consequently, they can be collected from flowers and leaves and then transferred to the hive. In turn, AIs that have deep-seated and systemic activity can be collected by bees together with pollen and nectar.

The AIs of PPPs, transferred by the worker bees to the hives, may result in miscellaneous, distinct effects. Łozowicka (2013) investigated cases of honeybee colony intoxication. A presence of cypermethrin (pyrethroid insecticide, detected in 51% samples), chlorpyrifos (organophosphorus insecticide, detected in 27% samples), and bifenthrin (pyrethroid insecticide, detected in 21% samples) was found in 33 worker bee samples. Likewise, in intoxicated worker bee bodies, Walorczyk and Gnusowski (2009) found an occurrence of tebuconazole (triazole fungicide, detected in 48% samples), omethoate (oxygenated form of dimethoate, organophosphorus insecticide, detected in 44% samples), and fipronil (phenylpyrazole insecticide, detected in 40% samples). In turn, among 19 of the detected compounds, Barganska et al. (2014) most frequently found heptenophos (organophosphorus insecticide, detected in 68% samples), bifenthrin (pyrethroid insecticide, detected in 53% samples), and pyrazophos and diazinon (organophosphorus insecticide, detected in 32% samples). However, cases of acute honeybee poisoning by the PPPs may be only marginal. In most of the cases, the presence of pesticides in the hive is at such a low level that it does not affect the honeybee colony well-being. Pohorecka et al. (2017) suggest that the phenomenon of winter colony collapse could be caused by honeybee parasites. Studies of Piechowicz et al. (2018a, b) on the transfer of plant protection products from oilseed rape crops and orchards to beehives showed a presence of pesticides both in bee bodies (5/7 detected compounds at rape plantation 1; 3/5 at rape plantation 2; and 5/6 AIs in orchards) and in honeybee brood (4 and 2 AIs in hives located near rape crops and 6 AIs in bees in the orchard), and in honey (3 and 3 AIs in rape honey and 4 AIs in apple-pear honey). In the studied cases, when the worker bees were directly exposed to pesticides originating from the crops, no deterioration in honeybee colony well-being was observed. It does not mean that PPP AIs, especially in the case of their simultaneous presence in the hive, could not have affected the bees. Some investigators indicate that for bees endowed with only 46 genes responsible for the detoxification system functioning (Claudianos et al. 2006), which additionally have few genes controlling detoxification of the plant protection products (The honeybee genome sequencing consortium 2006), a synergistic action of small, sublethal residues of two or more AIs (Thompson 1996; Thompson and Wilkins 2003; Mullin et al. 2010; Glavan and Božič 2013; Johnson et al. 2013) can be dangerous for them. Even if these compounds are not toxic to bees, this does not mean that they are not harmful to the brood (Zhu et al. 2014). This effect is especially relevant to intensively protected crops in which the flowering and fruiting periods occur at the same time, so both plants and fruits need protection. Raspberries are one such crop.

Our study was an initial analysis of whether some AIs of PPPs may be carried by bees from raspberry plants and transferred to beehives located in the immediate vicinity of the crop and to what extent these AIs were transferred.

## Materials and Methods

### Field Trial

The field trial was performed from May 20 to July 15, 2014, on a raspberry (*Rubus idaeus*), Laszka variety, plantation in the village Grabówka Kolonia, in the province of Lublin, which is protected from pests using conventional methods, in accordance with current programmes. All preparations were applied according to the labels posted. A sprayer, model RA 10/80 (Lochmann, Vilpiano, Italy) with nozzles ALBUZ ATR 80, was used. Within 2 km of the studied raspberry plantation, there were no plantations of any other blossoming plants secreting nectar that could have interfered with the test results. The honeybee colonies were transported from an area where the bees had no contact with pesticides. On May 17, 2014, the colonies were placed approximately 3 m from the raspberry plantation on an area of 4 ha.

On the raspberry plantation, four rows of plants were chosen for the study, each approximately 150 m long. On each sampling date, from each of the four selected rows, a sample of 16 leaves from randomly selected plants was taken (only fully developed leaves were collected from the outside of the bush), and then analytical portions, which consisted of 16 disks 1 cm in diameter, were cut. On the same sampling day, samples of flowers and fruits, consisting of 8 and 16 pieces, respectively, were collected from the same randomly selected plants.

During the field trial, from each of the four hives, one laboratory sample of worker bees (retrieved from the frames), the brood (from non-sealed cells, 4–6 days before hatching), and honey (from non-sealed cells) also were collected. Each sample weighed at least 5 g. Additionally, every week, soil samples were collected using an Egner stick, with one sample from each of the four rows. Each sample consisted of eight portions taken from randomly selected places in the row at a distance no further than 30 cm from the raspberry plants.

### Chemicals and Pesticides

During the period from January 7 to June 9, 2014, 12 protective treatments were performed on the plantation. The terms, preparations and applied doses are shown in Table 1.

**Table 1:** Pesticide spraying program performed on the raspberry plantation

Date of treatment	PPP, trade name	AI, common name (IUPAC name)	Chemical classification	AI structure	AI content	Mode of application	Mechanism of action	Dose of PPP (L, kg/ha)	MRL, honey (mg/kg)
January 7	Kerb 50 WP (H)*	Propyzamide (3,5-dichloro-*N*-(1,1-dimethylprop-2-ynyl)benzamide)	Benzonitrile		500 g/kg (50%)	Between rows	Systemic	2.00	0.05
March 23	Treol 770 EC (I)	Paraffin oil	–	–	770 g/L	Foliar	By contact—plants; By contact—insects	20.00	0.01
May 7	Amistar 250 SC (F)	Azoxystrobin (methyl (E)-2-{2-[6-(2-cyanophenoxy)pyrimidin-4-yloxy]phenyl}-3-methoxyacrylate)	Strobilurin		250 g/L (22.81%)	Foliar	Systemic	0.50	0.05
May 7	Score 250 EC (F)	Difenoconazole (1-[2-[2-chloro-4-(4-chloro-phenoxy)-phenyl]-4 methyl[1,3]dioxolan-2-ylmethyl]-1H-1,2,4-triazole)	Triazole		250 g/L (23.58%)	Foliar	Systemic	0.50	0.05
May 14	Mythos 300 SC (F)	Pyrimethanil (4,6-dimethyl-*N*-phenylpyrimidin-2-amine)	Anilinopyrimidine		300 g/L (28.3%)	Foliar	By contact and deep-seated	2.50	0.05
May 14	Dursban 480 EC*(I)	Chlorpyrifos (*O*,*O*-diethyl *O*-3,5,6-trichloropyridin-2-yl phosphorothioate)	Organophosphate		460 g/L (44.86%)	To the soil through the irrigation system	By contact and deep-seated—plants; By ingestion—insects	2.00	0.05
May 16	Mospilan 20 SP (I)	Acetamiprid (*N*-[(6-chloro-3-pyridyl)methyl]-*N’*-cyano-*N*-methyl-acetamidine)	Neonicotinoids		200 g/kg (20%)	Foliar	Systemic—plants; by ingestion—insects	0.20	0.05
May 19/May 29	Cyperkil Super 250 EC (I)	Cypermethrin ([cyano-(3-phenoxyphenyl)methyl]3-(2,2-dichloroethenyl)-2,2-dimethylcyclopropane-1-carboxylate)	Pyrethroids		250 g/L (25.92%)	Foliar	By contact—plants;	0.15	0.05
							By contact, by ingestion—insects	0.15	0.05
June 4	Rovral Aquaflo 500 SC (F)	Iprodione (3-(3,5-dichlorophenyl)-*N*-isopropyl-2,4-dioxoimidazolidine-1-carboxamide)	Dicarboximide		500 g/L (42.91%)	Foliar	By contact	2.00	0.05
June 5	Bellis 38 WG (F)	Boscalid (2-chlor-*N*-(4′-chlorbiphenyl-2-yl)nicotinamid)	Anilide		252 g/kg (25.2%)	Foliar	Systemic	1.50	0.50
		Pyraclostrobin (methyl-*N*-(2-[1-(4-chlorphenyl)-1*H*-pyrazol-3-yl]oxymethylphenyl)-(*N*-methoxy)carbamat)	Strobilurin		128 g/kg (12.8%)				0.05
June 9	Signum 33 WG (F)	Boscalid (2-chlor-*N*-(4′-chlorbiphenyl-2-yl)nicotinamid)	Anilide		267 g/kg (26.7%)	Foliar	Systemic	1.80	0.50
		Pyraclostrobin (methyl-*N*-(2-[1-(4-chlorphenyl)-1*H*-pyrazol-3-yl]oxymethylphenyl)-(*N*-methoxy)carbamat)	Strobilurin		67 g/kg (6.7%)				0.05

*H herbicide, *I* insecticide, *F* fungicide

### Extraction of Pesticide Residues from the Honey, Worker Bees, and Brood for Analysis

The samples of the worker bees and the brood were lyophilized using a Labconco Freezone 2.5 freeze dryer (Labconco, USA) (pressure: 0.024 mbar; temperature: 50 °C, time: 168 h).

Analytical portions of 5 g of the lyophilized animals or honey were shaken with 10 mL of acetonitrile (Chempur, Poland). Then, a mixture of salts containing 4 g of anhydrous magnesium sulfate (VI) (Chempur, Poland), 1 g of sodium chloride (Chempur, Poland), 1 g of trisodium citrate (Chempur, Poland), and 0.5 g of sesquihydrate disodium hydrogen citrate (Chempur, Poland) was added. The contents were shaken for 2 min and centrifuged for 5 min at 4500 rpm at 21 °C. Six millilitres of the acetonitrile phase was transferred to a polypropylene test tube that contained 150 mg of PSA (primary secondary amine) (Agilent, USA) and 900 mg of anhydrous sodium sulfate(VI) (Chempur, Poland). The extract was vigorously shaken for 2 min and centrifuged for 5 min as described above. Four millilitres of the obtained extract was taken and transferred to a glass tube, evaporated to dryness on a Heidolph Efficient Labware 4000 rotary evaporator (Heidolph, Germany), and then dissolved in 4 mL of petroleum ether (Chempur, Poland).

### Extraction of Pesticide Residues from the Leaves, Flowers and Fruits for Analysis

The analytical portions of the leaves (16 disks, 1 cm in diameter each) and flowers (8 pieces), both with the addition of 100 mL of water, and fruits (16 pieces) were homogenized in a Waring Commercial 8010 EG blender (Waring, USA) with 150 mL of acetone (Chempur, Poland) and filtered through a Büchner funnel under vacuum. The blender jar was flushed with 50 mL of acetone, and the washings were used to wash the filter cake. One-fifth of the filtrate (the equivalent of approximately 15.4 g of fruit and approximately 0.1 g of leaves) was used for further analysis. It was placed in a separatory funnel together with 100 mL of 2.5% sodium sulfate (VI) (Chempur, Poland) solution. The pesticide residues were extracted three times with 20, 10, and 10 mL of dichloromethane (Chempur, Poland). The combined extracts were evaporated to dryness, dissolved in 10 mL of petroleum ether and purified using a Florisil (Chempur, Poland) mini-column (Sadło et al. 2014, 2015). The pesticide residues were eluted with a 70-mL mixture of 3:7 (v/v) ethyl ether:petroleum ether (Chempur, Poland) as well as with a 70-mL mixture of 3:7 (v/v) acetone:petroleum ether. The solvents were evaporated to dryness, and the residue was transferred quantitatively using petroleum ether into a 10-mL volumetric measuring flask.

### Extraction of Pesticide Residues in the Soil for Analysis

The soil laboratory samples were air dried and pulverized with a Testchem LMG grinder (Testchem Sp. z.o.o., Poland) and stirred carefully. Analytical portions of 20 g were taken from the samples and shaken for 1 h with a 50-mL mixture of dichloromethane:acetone (9:1; v/v) on a GFL 3006 shaker (GFL, Germany). The extracts were allowed to stand for 10 min and then decanted through a layer of anhydrous sodium sulfate (VI) that had been placed in the funnel. The soil samples were washed twice with 20 mL of dichloromethane, and the combined extracts were evaporated to dryness on a Heidolph Laborota 4000 Efficient rotary evaporator. The residues were then dissolved in 10 mL of petroleum ether. The resulting extracts were purified using a Florisil mini-column (Sadło et al. 2014, 2015), and the residues were eluted with 70 mL of 3:7 (v/v) diethyl ether:petroleum ether, followed by elution with 70 mL of 3:7 (v/v) acetone:petroleum ether. The combined eluates were evaporated to dryness, and the residues were quantitatively transferred using petroleum ether into a 10-mL volumetric measuring flask.

### Chromatographic Determination of Pesticide Residues

The extracts were analysed using an Agilent 7890 (Agilent, USA) gas chromatograph equipped with a micro-cell electron capture detector (µECD) and a nitrogen–phosphorus detector (NPD). The chromatograph was controlled by ChemStation software (Agilent, USA). It also was equipped with an autosampler and an HP-5MS, 30-m × 0.32-mm × 0.25-µm column. The instrumental analysis conditions were as follows: an NPD detector temperature of 300 °C, a μECD detector temperature of 290 °C, and an injector temperature of 250 °C. The oven temperature was programmed as follows: 100 °C at 0 min → 10 °C per min → 4 min at 180 °C → 3 °C per min → 15 min at 220 °C → 10 °C per min → 11 min at 260 °C. The total analysis time was 55.3 min, and the injection volume was 2 µL.

### Statistical Analysis of the Results

Recovery studies were performed by spiking each matrix with the substances used in field trials at a single concentration (Table 2). The pesticide residues (*R*_*i*_) in the samples were recalculated (*R*_rec_) using the results of the recovery study (Rec in %; Table 3) according to Eq. 1.

**Table 2:** Recoveries (expressed in %) of AIs of PPPs applied on the raspberry plantation

Sample	Propyzamide	Chlorpyrifos	Iprodione	Pyraclostrobin	Boscalid	Cypermethrin	Difenoconazole	Azoxystrobin	Pyrimethanil
Worker bees	96.3	95.7	92.5	95.0	90.1	108.3	105.2	86.7	101.8
Brood	95.6	89.9	111.2	90.5	95.0	110.4	120.0	111.7	107.3
Soil	90.0	88.9	86.8	108.5	100.8	114.3	110.4	86.7	87.4
Honey	100.1	88.4	76.9	76.0	63.4	87.0	75.6	61.4	93.2
Flowers	94.2	92.4	98.5	117.4	89.0	85.7	93.2	82.2	93.2
Leaves	98.7	92.1	101.3	112.9	91.6	86.1	85.7	82.5	94.3
Fruits	92.0	91.9	88.2	118.1	85.1	85.7	85.3	81.6	93.7

**Table 3:** The average residues ± standard deviations (in mg/kg) of AIs applied on the raspberry plantation

Sampling date	Propyzamide	Chlorpyrifos	Iprodione	Pyraclostrobin	Boscalid	Cypermethrin	Difenoconazole	Azoxystrobin	Pyrimethanil
*Flowers*									
May 20	< LOQ	< LOQ	< LOQ	< LOQ	< LOQ	0.33 ± 0.21	< LOQ	< LOQ	24.50 ± 6.60
May 27	< LOQ	0.09 ± 0.04	< LOQ	< LOQ	< LOQ	0.24 ± 0.15	1.03 ± 0.15	0.14 ± 0.05	3.41 ± 1.02
June 4	< LOQ	< LOQ	< LOQ	< LOQ	< LOQ	0.51 ± 0.51	0.84 ± 0.54	< LOQ	1.72 ± 0.68
June 10	< LOQ	< LOQ	< LOQ	0.36 ± 0.27	7.20 ± 3.84	0.57 ± 0.09	< LOQ	< LOQ	1.75 ± 0.76
June 17	< LOQ	< LOQ	< LOQ	0.30 ± 0.11	6.34 ± 2.40	0.41 ± 0.03	< LOQ	< LOQ	0.03 ± 0.03
June 25	< LOQ	< LOQ	< LOQ	0.04 ± 0.04	1.94 ± 0.77	0.18 ± 0.11	< LOQ	< LOQ	< LOQ
July 2	< LOQ	< LOQ	< LOQ	< LOQ	< LOQ	0.63 ± 0.11	< LOQ	< LOQ	0.10 ± 0.03
July 8	< LOQ	< LOQ	< LOQ	0.08 ± 0.16	1.08 ± 2.16	0.51 ± 0.44	< LOQ	< LOQ	0.06 ± 0.07
*Leaves*									
May 20	< LOQ	0.13 ± 0.15	< LOQ	< LOQ	< LOQ	0.90 ± 1.27	< LOQ	< LOQ	206.28 ± 61.02
May 27	< LOQ	0.11 ± 0.22	< LOQ	< LOQ	< LOQ	0.11 ± 0.13	31.98 ± 8.09	5.61 ± 0.91	30.29 ± 17.85
June 4	< LOQ	< LOQ	< LOQ	< LOQ	< LOQ	2.80 ± 1.11	9.71 ± 2.40	1.39 ± 0.90	34.05 ± 25.18
June 10	< LOQ	< LOQ	< LOQ	12.30 ± 4.49	7.40 ± 1.60	1.54 ± 0.32	4.92 ± 0.48	< LOQ	2.50 ± 0.42
June 17	< LOQ	< LOQ	< LOQ	19.59 ± 7.52	7.61 ± 3.62	0.96 ± 0.44	7.20 ± 3.72	0.67 ± 0.67	3.25 ± 1.91
June 25	< LOQ	< LOQ	< LOQ	11.70 ± 4.34	5.51 ± 1.96	1.33 ± 0.33	4.17 ± 1.57	0.44 ± 0.44	5.44 ± 3.58
July 2	< LOQ	0.06 ± 0.06	< LOQ	6.49 ± 2.39	4.41 ± 1.40	1.20 ± 0.40	3.78 ± 0.53	< LOQ	0.27 ± 0.27
July 8	< LOQ	< LOQ	< LOQ	2.84 ± 1.26	2.23 ± 0.82	0.47 ± 0.10	1.54 ± 0.92	< LOQ	< LOQ
July 15	< LOQ	< LOQ	< LOQ	0.60 ± 0.35	1.11 ± 0.42	0.34 ± 0.09	2.09 ± 1.25	< LOQ	< LOQ
*Fruits*									
June 17	< LOQ	< LOQ	< LOQ	0.04 ± 0.01	1.34 ± 0.29	0.27 ± 0.07	0.02 ± 0.00	0.01 ± 0.00	0.16 ± 0.05
June 25	< LOQ	< LOQ	< LOQ	0.03 ± 0.00	0.84 ± 0.18	0.14 ± 0.05	0.01 ± 0.00	< LOQ	0.04 ± 0.00
July 2	< LOQ	< LOQ	< LOQ	< LOQ	0.41 ± 0.08	0.07 ± 0.02	0.01 ± 0.00	< LOQ	0.02 ± 0.00
July 8	< LOQ	< LOQ	< LOQ	< LOQ	0.23 ± 0.06	0.02 ± 0.00	0.01 ± 0.00	< LOQ	0.01 ± 0.00
July 15	< LOQ	< LOQ	< LOQ	< LOQ	0.05 ± 0.00	0.02 ± 0.00	0.01 ± .0.00.	< LOQ	0.01 ± 0.00
*Soil*									
May 20	0.16 ± 0.08	0.08 ± 0.08	< LOQ	< LOQ	< LOQ.	< LOQ	< LOQ	< LOQ	< LOQ
May 27	0.10 ± 0.05	0.06 ± 0.05	< LOQ	< LOQ	< LOQ	< LOQ	< LOQ	< LOQ	< LOQ
June 4	0.07 ± 0.03	0.04 ± 0.03	< LOQ	< LOQ	< LOQ	< LOQ	< LOQ	< LOQ	< LOQ
June 10	0.04 ± 0.02	0.03 ± 0.02	< LOQ	< LOQ	< LOQ	< LOQ	< LOQ	< LOQ	< LOQ
June 17	0.03 ± 0.01	0.02 ± 0.01	< LOQ	< LOQ	< LOQ	< LOQ	< LOQ	< LOQ	< LOQ
June 25	0.02 ± 0.01	0.01 ± 0.01	< LOQ	< LOQ	< LOQ	< LOQ	< LOQ	< LOQ	< LOQ
July 2	0.01 ± 0.01	0.01 ± 0.00	< LOQ	< LOQ	< LOQ	< LOQ	< LOQ	< LOQ	< LOQ
July 8	0.01 ± 0.00	0.01 ± 0.00	< LOQ	< LOQ	< LOQ	< LOQ	< LOQ	< LOQ	< LOQ
July 15	0.01 ± 0.00	0.01 ± 0.00	< LOQ	< LOQ	< LOQ	< LOQ	< LOQ	< LOQ	< LOQ
*Honey*									
May 20*	< LOQ	< LOQ	< LOQ	< LOQ	< LOQ	< LOQ	< LOQ	< LOQ	< LOQ
May 27	< LOQ	< LOQ	< LOQ	< LOQ	< LOQ	< LOQ	< LOQ	< LOQ	< LOQ
June 4	< LOQ	< LOQ	< LOQ	< LOQ	< LOQ	< LOQ	< LOQ	< LOQ	< LOQ
June 10	< LOQ	< LOQ	< LOQ	< LOQ	0.01 ± 0.00	< LOQ	< LOQ	< LOQ	< LOQ
June 17	< LOQ	< LOQ	< LOQ	< LOQ	< LOQ	< LOQ	< LOQ	< LOQ	< LOQ
June 25	< LOQ	< LOQ	< LOQ	< LOQ	< LOQ	< LOQ	< LOQ	< LOQ	< LOQ
July 2	< LOQ	< LOQ	< LOQ	< LOQ	< LOQ	< LOQ	< LOQ	< LOQ	< LOQ
July 8	< LOQ	< LOQ	< LOQ	< LOQ	< LOQ	< LOQ	< LOQ	< LOQ	< LOQ
July 15	< LOQ	< LOQ	< LOQ	< LOQ	< LOQ	< LOQ	< LOQ	< LOQ	< LOQ
*Worker honeybees*									
May 20	< LOQ	0.03 ± 0.00	< LOQ	< LOQ	< LOQ	< LOQ	< LOQ	< LOQ	0.09 ± 0.03
May 27	< LOQ	0.03 ± 0.00	< LOQ	< LOQ	< LOQ	< LOQ	< LOQ	< LOQ	0.03 ± 0.01
June 4	< LOQ	0.05 ± 0.01	< LOQ	< LOQ	< LOQ	< LOQ	< LOQ	< LOQ	< LOQ
June 10	< LOQ	0.04 ± 0.01	0.09 ± 0.09	< LOQ	0.08 ± 0.05	< LOQ	< LOQ	< LOQ	< LOQ
June 17	< LOQ	0.01 ± 0.04	0.11 ± 0.13	< LOQ	0.06 ± 0.10	< LOQ	0.02 ± 0.07	< LOQ	< LOQ
June 25	< LOQ	0.05 ± 0.00	0.63 ± 0.16	< LOQ	0.11 ± 0.05	< LOQ	< LOQ	< LOQ	< LOQ
July 2	< LOQ	0.02 ± 0.01	0.01 ± 0.01	< LOQ	0.01 ± 0.01	< LOQ	0.12 ± 0.03	< LOQ	< LOQ
July 8	< LOQ	0.02 ± 0.01	0.02 ± 0.02	< LOQ	0.01 ± 0.01	< LOQ	0.09 ± 0.03	< LOQ	< LOQ
July 15	< LOQ	< LOQ	< LOQ	< LOQ	< LOQ	< LOQ	< LOQ	< LOQ	< LOQ
*Worker brood*									
May 20	< LOQ	0.01 ± 0.01	< LOQ	< LOQ	< LOQ	< LOQ	< LOQ	< LOQ	< LOQ
May 27	< LOQ	0.02 ± 0.01	< LOQ	< LOQ	< LOQ	< LOQ	< LOQ	< LOQ	< LOQ
June 4	< LOQ	< LOQ	< LOQ	< LOQ	< LOQ	< LOQ	< LOQ	< LOQ	< LOQ
June 10	< LOQ	0.01 ± 0.01	< LOQ	< LOQ	< LOQ	< LOQ	< LOQ	< LOQ	< LOQ
June 17	< LOQ	0.01 ± 0.01	< LOQ	< LOQ	< LOQ	< LOQ	< LOQ	< LOQ	< LOQ
June 25	< LOQ	< LOQ	< LOQ	< LOQ	< LOQ	< LOQ	< LOQ	< LOQ	< LOQ
July 2	< LOQ	< LOQ	< LOQ	< LOQ	< LOQ	< LOQ	< LOQ	< LOQ	< LOQ
July 8	< LOQ	< LOQ	< LOQ	< LOQ	< LOQ	< LOQ	< LOQ	< LOQ	< LOQ
July 15	< LOQ	< LOQ	< LOQ	< LOQ	< LOQ	< LOQ	< LOQ	< LOQ	< LOQ

*In worker honeybees and the brood as well in honey samples collected on May 16 (a day before transporting hives to the crop), none of the determined PPP AIs were shown

1 $$R_{\text{rec}} = 100 \, \times \, R_{i} /{\text{Rec}}$$

The transfer factor (TF) from the soil to the plants was calculated according to Eq. 2.

2 $${\text{TF}} = 100 \times R_{{i\,({\text{plant}})}} /R_{{i\,({\text{soil}})}}$$

To determine the similarity in the residue concentrations of the individual substances between different sample types, a cluster analysis was performed. The Euclidean metric was used to describe the similarities. The Ward method was used as the agglomeration algorithm. The Friedman test was used to determine whether the residue concentrations in the sample types varied significantly. The Spearman’s rank correlation coefficient was used to assess the strength, direction and statistical significance of dependencies between the residue concentrations in the various sample types, assuming *α* < 0.05 as statistically significant.

## Results

In general, pesticide residue recoveries should be in the range of 70–120% of the substance introduced into the sample, and the repeatability should be ≤ 20% (Document SANTE 2015). In our study, satisfactory values of both of these parameters were obtained for nine AIs of PPPs in seven sample types. However, for boscalid and azoxystrobin, the recovery from honey did not exceed 70%; the recoveries were 63.4 and 61.4%, respectively (Table 2). The limit of quantification (LOQ) of propyzamide, chlorpyrifos, iprodione, pyraclostrobin, boscalid, cypermethrin, difenoconazole, azoxystrobin, and pyrimethanil in all studied matrices was 0.01 mg/kg.

### Pesticide Residues in Raspberry Plantation

Table 3 shows the average concentrations of pesticide residues in the seven sample types.

#### Flowers

Seven of the nine studied AIs were found in the flowers. The residues of chlorpyrifos (an organophosphorus insecticide with a deep-seated mode of action in plants) were applied to the soil by the irrigation system on May 14 in the form of Dursban 480 EC at an application rate of 2 L/ha, and azoxystrobin (a strobilurin fungicide with a systemic mode of action in plants) was applied by foliar spraying on May 7 in the form of Amistar 250 SC at an application rate of 0.5 L/ha. The residues of those substances were found in only the samples collected on May 27 (13 and 20 days since application) at concentrations of 0.09 and 0.14 mg/kg of the flowers, respectively. The TF from the soil to flowers for chlorpyrifos was 150%, which indicated that a distinct part of that substance penetrated the flowers from the soil with the transpiration current. On only two sampling days, in the samples collected on May 27 and June 4, difenoconazole residues (triazole fungicide with a systemic mode of action) were found at a relatively high level (1.03 and 0.84 mg/kg). This substance was applied on May 7 in the form of Score 250 EC (0.5 L/ha). Pyrimethanil, which was applied in the form of Mythos 300 SC at an application rate of 2.5 L/ha on May 14, occurred at the highest level. The initial residue of this compound, with slight fluctuations in the levels, decreased in concentration until the last sampling day. Boscalid (anilide fungicide) and pyraclostrobin (strobilurin fungicide) residues, after the application of Bellis 38 WG (1.5 kg/ha) on June 5 and Signum 33 WG (1.8 kg/ha) on June 9 (both preparations with a systemic mode of action in plants), were found on four sampling days, and their values gradually decreased, excluding the samples collected on June 2, when none of the two AIs was found, from 7.2 to 0.36 mg/kg in the flower samples collected on June 10 to 1.08 and 0.08 mg/kg in the flower samples from July 8. Cypermethrin (pyrethroid insecticide with a contact mode of action) was applied on May 19 and May 29 in the form of Cyperkill Super 250 EC, in both cases at an application rate of 0.15 L/ha. Its residues were observed in the flower samples collected on all sampling days, while after the first application (samples from May 20), its residues were 0.33 mg/kg, and after the second application, they were 0.51 and 0.57 mg/kg, respectively. From that time until June 25, the concentrations decreased to 0.18 mg/kg and then increased to the 0.63 mg/kg on July 2.

#### Leaves

In the laboratory leaf samples, chlorpyrifos, pyraclostrobin, boscalid, cypermethrin, difenoconazole, azoxystrobin, and pyrimethanil were found. Chlorpyrifos residues were detected on May 20 and May 27 (0.13 and 0.11 mg/kg, respectively) and on July 2 (0.06 mg/kg). In this case, the TF was 162.5, 183.3, and 600%, respectively. Pyraclostrobin and boscalid residues were found in samples collected on six sampling days, and their highest values occurred on June 17 (19.59 and 7.61 mg/kg, respectively). After that, since the last sampling day, a systematic decrease in their level was observed. It is worth mentioning that the residues of pyraclostrobin were significantly higher than those of boscalid, which was in contradiction to proportions of those AIs in the applied preparations (1:1.97 in the case of Bellis 38 WG and 1:3.99 in the case of Signum 33 WG). The cypermethrin residues were detected in all the samples, while after the first application of Cyperkill Super 250 EC, they were 0.9 mg/kg (samples collected on May 20), and after the second application, they were 2.8 mg/kg (samples collected on June 4). From that time until the end of the investigation, a decrease in the cypermethrin residue level was observed. Likewise, in all samples, excluding those collected on May 20, the presence of the applied difenoconazole was found; its residues appeared to be high (31.98 mg/kg) on May 27; and after that day, they decreased until the last sampling day (2.09 mg/kg). Azoxystrobin was detected in samples collected on four application days. Its highest residue level was found in leaves collected on May 27 (5.61 mg/kg), and in subsequent application dates, this value decreased to 0.44 mg/kg in the samples collected on June 25, while the residues of this AI were not found in the samples from June 10. After foliar application on May 14, pyrimethanil left significant residues on leaves (206 mg/kg in the samples from May 20) that decreased to 0.27 mg/kg in the samples from July 2 and to an amount < LOQ during the subsequent leaf sampling days.

#### Fruits

The harvest period for fruits began on June 17, and pyraclostrobin, boscalid, cypermethrin, difenoconazole, pyrimethanil, and azoxystrobin were detected at that time. The pyraclostrobin residues were found only at a level 3–4 times higher than the limit of quantification (LOQ) on June 17 and June 25 (0.04 and 0.03 mg/kg, respectively), while boscalid, applied together with pyraclostrobin, in all samples, ranged from 1.34 mg/kg on June 17 to 0.05 mg/kg on July 15. The cypermethrin residues were found in all samples of fruit, and its residue decreased from 0.27 mg/kg on June 17 to 0.02 mg/kg on the last sampling day. Similarly, difenoconazole and pyrimethanil were found in fruit samples on each sampling date, while in the case of difenoconazole, its residues occurred at the level closest to the LOQ (0.02 mg/kg on June 17 and 0.01 mg/kg on other dates), and the pyrimethanil residue level decreased with time from 0.16 mg/kg on June 17 to 0.01 mg/kg on June 15. Trace azoxystrobin residues were found only in samples collected on June 17 (0.01 mg/kg).

#### Soil

Only two AIs of PPPs applied were detected in the 10-cm soil layer. In all studied samples, the residue of chlorpyrifos decreased systematically from 0.08 mg/kg of soil on May 20 to 0.01 mg/kg in the samples collected from June 25 to July 15, and propyzamide, with an AI of Kerb 50 WP, belonging to benzonitrile group. The herbicide was applied between rows on January 7 at an application rate of 2 L/ha, and its residues decreased systematically from 0.16 mg/kg on the first sampling day to 0.01 mg/kg in the samples collected during the period July 2 to July 15.

### Pesticide Residues in Samples of Honey, Worker Bees and Brood

#### Honey

Only a trace residue of boscalid at the level of the LOQ (0.01 mg/kg on June 10) was detected in honey on one sampling date.

#### Worker Bees and Brood

Chlorpyrifos, iprodione, boscalid, difenoconazole, and pyrimethanil were found in worker bee samples. Chlorpyrifos residues were detected most frequently and were found in samples taken for analysis on eight of nine sampling dates (May 20 and 27; June 4, 10, 17, and 25; and July 2 and 8), although in all cases, its residues were at low levels (to 0.05 mg/kg in the samples collected on June 4). However, animal bodies contained iprodione residues (dicarboximide group; the AI of Rovral Aquaflo 500 SC, used on June 4 at an application rate of 2 L/ha), a fungicide with a contact mode of action on plants, which was not detected in other matrices. The iprodione residues increased during the period from June 10 to June 25 from 0.09 to 0.63 mg/kg and then started to decrease below LOQ on the last sampling day. The boscalid residues were detected at low levels (no more than 0.11 mg/kg on June 25), but pyraclostrobin, which was applied in the form of Bellis 38 WG and Signum 33 WG together with boscalid, was not detected at all. Similarly, in the worker bee samples, small amounts of systemic difeconazole were detected (in the samples from 3 sampling days; to 0.12 mg/kg in samples from July 2) as well as pyrimethanil with a contact and deep-seated action (0.09 and 0.03 mg/kg in the samples from May 20 and May 27, respectively).

In brood samples, the pesticide residues at a level higher than the LOQ were collected only on three sampling days. Only a trace amount of chlorpyrifos was found (no more than 0.02 mg/kg of the brood) on May 27.

### Statistical Analysis

The similarity degree for the residue concentrations of the individual substances found in the various sample types, regardless of the sampling date, was assessed using a cluster analysis. Based on the cluster analysis results and the associated distance matrix analysis, differences in the residue concentrations of the individual substances could be seen in the various sample types. However, when using the Friedman test, statistically significant differences between the residue concentrations of specific substances were not demonstrated.

Spearman’s rank correlation coefficient was used to determine whether there was a mutual relationship between the residue concentrations in the samples analysed, regardless of the collection date (Table 4).

**Table 4:** The relationship between residue concentrations found in different matrix combinations

AI, common name	Matrix combination	Correlation level
Chlorpyrifos	Soil-worker brood	0.75
Iprodione	All	Lack of relationship
Pyraclostrobin	All	Lack of relationship
Boscalid	Flowers-honey	0.80
	Flowers-worker honeybees	0.88
	Leaves-honey	0.87
	Leaves-worker honeybees	0.89
	Honey-worker honeybees	0.88
Cypermethrin	All	Lack of relationship
Difenoconazole	All	Lack of relationship
Azoxystrobin	All	Lack of relationship
Pyrimethanil	Flowers-worker honeybees	0.76

Spearman’s rank correlation coefficient was positive in all cases, which indicated a quantitative relationship between the application rate and the concentration of the residues of AIs of the PPP, located at sites frequented by the worker bees and in the hive interior.

Spearman’s rank correlation coefficient was also used to determine whether a mutual relationship existed between the residue concentrations of the AIs of PPPs in different combinations of samples and for different sampling conditions, and the results of the analysis of these relationships are presented in Table 5.

**Table 5:** The relationship between the residue levels of AIs of PPPs in various matrices, depending on terms of sampling

AI, common name	Sampling date	Relation	Correlation coefficient
Propyzamide	All	All	Lack of relationship
Chlorpyrifos	May 27	Leaves-worker honeybees	− 0.88
Iprodione	June 17	Soil-worker honeybees	− 0.89
Pyraclostrobin	All	All	Lack of relationship
Boscalid	June 10	Leaves-honey	0.81
	June 10	Leaves-worker honeybees	− 0.90
	June 25	Leaves-worker honeybees	0.82
	July 2	Flowers-honey	0.90
	July 2	Flowers-worker honeybees	0.87
	July 2	Fruits-worker honeybees	0.99
Cypermethrin	All	All	Lack of relationship
Difenoconazole	All	All	Lack of relationship
Azoxystrobin	All	All	Lack of relationship
Pyrimethanil	May 27	Flowers-worker honeybees	− 0.88

In most cases, a positive correlation was noted between the residue concentration of AIs of PPPs in the crop and in the hive. Only certain worker bees collected on May 27, June 10, and July 17 showed negative correlation coefficients. No positive correlations were noted between the AI of PPP residues in the environment and in the worker bees.

## Discussion

Raspberries, including the Laszka variety, are nectar-secreting plants. Their yields depend largely on the efficiency of their pollination by insects (Chauzat et al. 2009). Worker bees, when foraging on entomophilous plants, may simultaneously collect various contaminants and transfer them to the hive (Anderson and Wojtas 1986; Chauzat et al. 2009; Cresswell and Thompson 2012; Oruc et al. 2012; Piechowicz et al. 2018a, b). Some pesticides used to protect raspberry plantations from pests and diseases show a possibility of accumulation in the bee bodies. They also pollute bee products (Rissato et al. 2006). Therefore, some reports indicate that some AIs of PPPs may be transferred into the beehive (Anderson and Wojtas 1986; Southwick and Southwick 1992; Pettis et al. 2004; Panseri et al. 2014).

The AIs of PPPs differ in their chemical structure (they belong to different chemical groups) and mode of actions, which determine how they are distributed in the environment, how they spread in plants and penetrate plants, and how they penetrate animal bodies. Furthermore, the PPPs are characterized by long half-lives and consequently by their persistence in the environment (Gerolt 1983; Różański 1992; Leroux 1996; Mileson et al. 1998; Seńczuk 2012; Szpyrka and Walorczyk 2017). The above-mentioned factors may result in the occurrence of chlorpyrifos (deep-seated and probably, which is indicated by the results in Table 3, semi-systemic), azoxystrobin or difenoconazole residues (both with a systemic action in plants), which were not detected in the samples collected on May 20 (all 3 compounds; 6, 13, and 13 days after application, respectively), nor the leaves (azoxystrobin and difenoconazole). They were present only in samples collected on May 27, which indicates that their residues are linked to the secondary absorption of those compounds from the deeper layers of the soil, where they were probably diluted due to the intensive rainfalls rather than their primary presence at the plant surface. In addition, because only chlorpyrifos, at an application rate of 0.08 mg/kg on May 20 and of 0.06 mg/kg on May 27, was observed in the surface layer. As Kubik et al. (2000) notes, plant protection products with systemic action appear in the aboveground parts of plants at approximately 5 days after their introduction to the soil, which may partially explain the obtained results. The cypermethrin behaviour, which was applied in the form of Cyperkill Super 250 EC on May 19 and May 29, indicates the impacts of intensive rainfall on May 19 and May 20. On May 20, the first day after the first application, the cypermethrin residue on leaves was 0.9 mg/kg. In the samples obtained on June 4, i.e., 6 days after the second treatment (in both cases, 0.15 L/ha), this value increased to 2.8 mg/kg; therefore, without taking into account natural disappearance, it was threefold higher than after the first treatment.

Pyrimethanil, which was applied to the crop in the form of Mythos 300 SC at the same time as chlorpyrifos (Dursban 480 EC), i.e., on May 14, and cypermethrin, used in the form of Cyperkill Super 250 EC on May 19 and May 29, which are substances with contact action and more adherent to the plant surface, were found on leaves and flowers since the first sampling day (24.5 mg/kg on flowers and 206 mg/kg on leaves in the case of pyrimethanil and 0.33 mg/kg on flowers and 0.90 mg/kg on leaves in the case of cypermethrin). However, the residue of iprodione (AI of Rovral Aquaflo 500 SC), also with a contact mode of action, was not detected on any part of plant, although probably in this second case, the preparation, which was still damp, was washed away from the plant, which is partly indicative of the necessity of the subsequent fungicide treatment (Bellis 38 WG) that was applied on June 5, i.e., the day after the application of Rovral Aquaflo 500 SC. Most likely, not the lack of application but washing away the preparation from the plant resulted in the presence of marked amounts of iprodione on bee bodies, because the fungicide treatments were performed during the daily period of worker bee foraging.

Small amounts of difenoconazole and pyrimethanil at a level close to LOQ were observed on all fruit samples. Additionally, the presence of azoxystrobin was detected on June 17, which indicates that preparation of systemic and extensive action can occur in a plant for a long period (69, 62, and 41 days after application, respectively).

In the preparations, which were applied on June 5 and June 9 and contained pyraclostrobin and boscalid (Bellis 38 WG and Signum 33 WG), the proportions between these substances were 1:1.97 and 1:3.99, respectively. The amount of pyraclostrobin was reduced relative to the amount of boscalid that occurred in flowers (proportions from 1:13.5 on July 8 to 1:48.5 on June 25, respectively) and in fruits (1:33.5 on June 17 and 1:28.0 on June 25); however, in the case of leaves, we observed a complete reversal of the proportion, excluding samples from July 15 (the proportion pyraclostrobin to boscalid 1:1.85), in plants for a long period (69, 62, and 41 days after application, respectively). In the remaining sampling days, a larger residue of pyraclostrobin than boscalid (to 1:0.37 July 2 and July 8) was noted. The reversal of those proportions in the case of leaves may result from the increased transpiration of boscalid by the leaves compared with that of pyraclostrobin. Such a phenomenon was observed in the raspberry crop, for the pesticide residues from the surface of leaves, flowers, and fruits (material in preparation) when analysed exclusively.

Our surveys confirmed the possibility of transferring measurable (i.e., above the LOQ) amounts of some PPP AIs from the sprayed dessert raspberry bushes to the beehives. Most often, residues were found in worker bees; for example, chlorpyrifos was found in samples from eight of total nine sampling dates (from May 20 to July 8), iprodione from five of six sampling dates (from June 10 to July 8), boscalid from five of six dates (from June 10 to July 8), difenoconazole from three of nine dates (on June 17, July 2 and 8), and pyrimethanil from two of nine sampling dates (on May 20 and 27). Residues were found less frequently in the honey and brood and were found in only samples on one of eight sampling dates. Chlorpyrifos was found in broods from four of nine sampling dates (Tables 1, 3). The larger content of the pesticides in the worker bee bodies is a result of direct contact of the bee worker foragers with the sprayed plants, as well as their direct contact with PPPs, because the treatments were performed during the daily period of bee active foraging.

Preparations with deep-seated and systemic action on plants more frequently reached the hives than those with contact action. Chlorpyrifos, an organophosphorus insecticide and the AI of Dursban 480 EC, which was included in our study for the control of May bug larvae (*Melolontha melolontha*), is an example of the abovementioned rule. Unlike the other PPPs for which a foliar application was used, Dursban 480 EC was applied to the soil only via the irrigation system, and it had no direct contact with the aboveground parts of plants, on which foraging bees might be found. However, its long persistence in the soil (Table 3) and its continuous transport and concurrent transpiration to aboveground plant parts resulted in its occurrence in the flowers at a higher concentration than the LOQ, which consequently constituted a threat to pollinators. The presence of some pesticides in brood indicates that the worker bees have been exposed to PPPs since the earliest stages of their ontogeny, at a time of intensive nervous system development.

Chlorpyrifos, applied to the soil on May 14, was present in the soil samples until the last sampling date (i.e., July 8) and consequently also occurred in the flowers and leaves. The highest amounts of chlorpyrifos residues, compared with its residue in the soil, were detected on July 2 in fully developed leaves (TF = 600%), whose surfaces and weights did not change, which could suggest that this compound was still actively transported from the ground by the plant root system. In flowers, these residues were observed on May 27 (TF = 150%).

Iprodione, the fungicide with a contact mode of action, was found in worker bees (up to 0.63 mg/kg found in the sample on June 25), but the residue concentrations in the leaves, flowers, and fruits did not exceed the LOQ. An explanation of this effect may be the temporal bioaccumulation of this substance in animal tissues or foraging by bees on other crops. However, in a 2-km area around the crop, no other nectar-secreting blossoming plants were present, and such a large pesticide residue concentration would point to the transfer of large amounts of pesticides from other crops, where the preparation was applied between June 4 and June 10, when it was first observed in bee samples. It seems to be more likely, as it was mentioned above, that the preparation was used during daily period of the bee foraging, shortly before intense rainfall, which could remove the preparation from plants and top layer of the soil. However, it is worth mentioning that close to the studied plantation, there were small home cultivations of plants that did not secrete nectar, so they were unlikely to be attractive to bees (*Lycopersicon* Mill. or *Cucumis* L.); however, they were protected using preparations containing iprodione and constituted a place where the bees could stop, for example, to get water.

Despite the fact that various concentrations of PPP AI residues were found in the samples, it was not easy to estimate the significance of the differences between the concentrations. When the sampling conditions were not considered, a positive correlation between the presence of boscalid, pyrimethanil and chlorpyrifos in the crop and in the hive (Table 4) was found, which meant that an increase in the residues of AIs of PPPs in the beehive was linked to their higher concentrations in the crop. A similar relationship was observed when the sampling dates were considered (Table 5), although for chlorpyrifos (sampled on May 27), iprodione (sampled on June 1), boscalid (sampled on June 10), and pyrimethanil (sampled on May 27), negative correlations also were found, indicating that the increases in the AI residues in the crops were related to lower concentrations in the worker bee tissues. A probable reason for this phenomenon could have been the relatively intense and long rainfall that limited foraging intensity (personal communication from the crop owner).

Our studies were limited to 2 months. During this time period, no clear-cut declines in the strength of the tested honeybee colonies were observed. However, as previously mentioned, some AIs might be toxic to honeybees even at sublethal doses (Weick and Thorn 2002; Williamson and Wright 2013). As Leonardi et al. (1996) discusses, the AIs of pesticides even at a level lower than the LOQ can affect insects. They can act synergistically with each other (Thompson 1996; Thompson and Wilkins 2003, Glavan and Božič 2013), e.g., via competition for metabolic enzymes (Johnson et al. 2009) or cellular efflux (Hawthorne and Dively 2011) and with other environmental stressors (Renzi et al. 2016; Doublet et al. 2015). Thompson (1996) suggested that even PPPs considered safe for bees could intensify their activity against those insects by two orders of magnitude when used in combination with other PPPs.

The honeybees in the present study were exposed to four insecticides: paraffin oil, acetamiprid, chlorpyrifos, and cypermethrin. Paraffin oil physically disturbs the gas exchange process in pests (Card of Characteristics the preparation Treol 770 EC). Acetamiprid, being the agonist of nicotine acetylcholine receptors in the synapse, influences survival, including impairment of learning and memory, disruption of the navigation, and reduction of the honeybee foraging activity (Belzunces et al. 2012; Blacquiere et al. 2012; Henry et al. 2012). Acetamiprid significantly impairs olfactory learning in laboratory-based studies (Decourtye et al. 2004; Han et al. 2010). The next AI, chlorpyrifos, blocks the active sites of acetylcholinesterase in the synapse space by phosphorylation, and as a consequence, it intensifies the action of acetylcholine, which is distributed by chlorpyrifos, and influences honeybee learning and memory abilities in sublethal doses (Guez et al. 2010). Finally, cypermethrin causes elongation of sodium channel opening states in insect nervous cells (Wang and Wang 2003). It also is an agonist of the T-type calcium channel in insect muscles and is involved in the excretion of acetylcholine and dopamine in the synaptic space (Aldridge 1990). Moreover, it inhibits the mitochondrial complex I (Gassner et al. 1997), disrupts the protein phosphorylation process, and modifies the function of the gap junctional protein. Among the abovementioned insecticides, the presence of chlorpyriphos was detected in the worker bee bodies and brood. Unfortunately, the presence of acetamiprid (AI of Mospilan 20 SP, neonicotinoid insecticide applied on May 15) was impossible to determine due to poor recovery, but it would be interesting if, as suggested Kessler et al. (2015), honeybees prefer a diet containing neonicotinoids.

Bees in the crop also were exposed to six AIs of fungicides (i.e., azoxystrobin, difenoconazole, pyrimethanil, iprodione, boscalid, and pyraclostrobin), from which iprodione, boscalid, difenoconazole, and pyrimethanil were detected in worker bee bodies and boscalid was detected in honey. Additionally, pesticide adjuvants, which increased the probability of adverse interactions (Mullin et al. 2015) were found. Furthermore, because of the possibility of AI accumulation in the wax (Serra-Bonvehí and Orantes-Bermejo 2010), the adverse effects of those substances might extend far beyond the raspberry flowering period.

Even though chlorpyrifos, iprodione, boscalid, difenoconazole, and pyrimethanil were found in the bees and chlorpyrifos was found in the brood, a small amount of boscalid (0.01 mg/kg of honey) was detected in the honey on one sampling date, i.e., in samples collected on June 10. The amount of boscalid was so small that it did not exceed the MRL of 0.05 mg/kg (EU Pesticides Database 2017). The results indicate that honey from the beehive adjacent to the raspberry plantation, protected against pests and diseases, was a completely safe product in terms of the presence of the nine studied plant protection products.

Our surveys confirmed the possibility of transferring measurable amounts of some PPP AIs from the sprayed dessert raspberry bushes to the beehives. Five of the nine applied were detected in worker bee bodies. The honeybee brood was polluted by small amounts of chlorpyrifos applied to only the soil through the irrigation system. Only trace amounts of boscalid residues were detected in honey, which indicated that it was completely safe for consumption. The obtained results confirm occurrence of the phenomenon of active transferring the active ingredients of plant protection products (PPP AIs) by the honeybees from the crops to bee hives.
